# Investigation of the Effects of Length to Depth Ratio on Open Supersonic Cavities Using CFD and Proper Orthogonal Decomposition

**DOI:** 10.1155/2013/810175

**Published:** 2013-06-18

**Authors:** Ibrahim Yilmaz, Ece Ayli, Selin Aradag

**Affiliations:** Department of Mechanical Engineering, TOBB University of Economics and Technology, Sogutozu Cad., No. 43, 06560 Ankara, Turkey

## Abstract

Simulations of supersonic turbulent flow over an open rectangular cavity are performed to observe the effects of length to depth ratio (*L/D*) of the cavity on the flow structure. Two-dimensional compressible time-dependent Reynolds-averaged Navier-Stokes equations with k-*ω* turbulence model are solved. A reduced order modeling approach, Proper Orthogonal Decomposition (POD) method, is used to further analyze the flow. Results are obtained for cavities with several *L/D* ratios at a Mach number of 1.5. Mostly, sound pressure levels (SPL) are used for comparison. After a reduced order modeling approach, the number of modes necessary to represent the systems is observed for each case. The necessary minimum number of modes to define the system increases as the flow becomes more complex with the increase in the *L/D* ratio. This study provides a basis for the control of flow over supersonic open cavities by providing a reduced order model for flow control, and it also gives an insight to cavity flow physics by comparing several simulation results with different length to depth ratios.

## 1. Introduction

In several flow applications, especially for aerospace industry, unsteady, turbulent, and complex flow phenomenon becomes an important part of processes. In aeronautics applications, interior storage carriages, which are used to carry items such as weapons and bombs, are all cavity configurations. As high speed flows pass over cavities, a complex and unsteady flow field emerges in the cavity region. These flow fields lead to pressure fluctuations and relatively high sound pressure levels. Due to the pressure fluctuations and resonant acoustic modes, the flow passing over the cavity can damage the structure of air vehicles and impede successful store release as discussed by Aradag [1].

**Figure 1:**
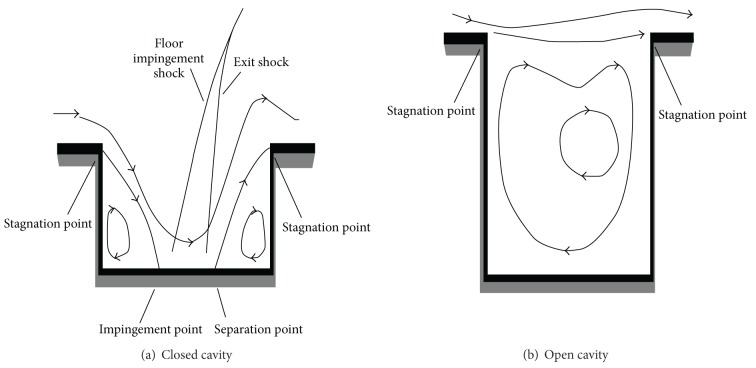
Supersonic cavity flow: (a) closed cavity, (b) open cavity.

The importance of the cavity flow mechanism resulted in many studies including some definitions and classifications about cavity flow. Due to the complexity of the flow mechanism in different cavity configurations, cavity flows are categorized based on mainly geometric specifications (*L*/*D* ratio, *L*/*W* ratio), cavity flow phenomena, and Mach number as discussed by Syed [2]. Geometric specifications of the cavity region affect the flow propagation in the cavity. When the flow is in the shallow cavity and *L*/*D* ratio is greater than 13, it is called closed cavity, whereas if the flow is in deep cavity and *L*/*D* ratio is smaller than 10, it is called open cavity configuration as discussed by Aradag [1]. As given in Figure 1, in open cavities, shear layer coming with the free-stream separates at the cavity leading edge and reattaches at the cavity back wall. Due to the shear layer formation, flows inside and outside the cavity are separated. Additionally, the pressure differences between inside and outside of the cavity lead to a single recirculation region inside the cavity. On the other hand, in closed cavity, after the separation of the shear layer, due to containing inadequate energy to pass the cavity, it impinges to cavity base then separates from the base and reattaches at the stagnation point at the trailing edge (Aradag [1]; Lawson and Barakos [3]). As seen in Figure 1, two flow fields are emerged inside the cavity due to the shear layer movements.

Numerous research studies have been performed on supersonic cavity flow to understand the cavity flow mechanism clearly. A pioneering study is performed by Rossiter [4], and an empirical formula to predict the frequencies of the pressure oscillation modes is defined. Heller and Bliss [5] modified this empirical formula to use it in all subsonic, supersonic, and transonic flows conditions. Shieh and Morris [6] used URANS Spalart-Allmaras turbulence model in their 2D and 3D cavity flow simulations. They examined vorticity contours for one period, and a periodic vortex formation in the cavity is observed. In their 3D simulation, the observed vortex is weaker than the one observed in 2D. Shih et al. [7] used *k*-*ε* turbulence model at Mach number of 1.5 and *L*/*D* ratio of 5.07 open cavity problem. They concluded that the shear layer formation makes the cavity flow more complex due to the mass input and output to the cavity. Rizzetta [8] performed 3D RANS simulations of cavity flow at a Mach number of 1.5. The results of average static pressure and sound pressure levels show good agreement with the experimental ones. In another study, Zhang and Edwards [9] used *k*-*ω* turbulence model to solve RANS equations for Mach numbers of 1.5 and 2.5 in different cavity geometries with *L*/*D* ratios of 1, 3, and 5. In conclusion, while the cavity flow is more uniform in *L*/*D* ratio of 1 cavity, when above the *L*/*D* ratio of 3, the flow becomes irregular and oscillations frequencies are increased. These numerical studies include RANS equations. There are also other studies using Large Eddy Simulations (LES) and Detached Eddy Simulation (DES) to perform cavity flow simulations. Hamed et al. [10] used DES method to model supersonic cavity flow with *L*/*D* of 5 and Mach number of 1.19. Basu et al. [11], Barakos et al. [12], and Rizzetta [8] also performed simulations on supersonic cavity flow.

Besides the numerical studies, there are experimental studies about supersonic cavity flow. Bueno et al. [13] experimentally investigated the effects of *L*/*D* ratio on the cavity flow, and according to their results, when cavity length increases, pressure fluctuations also increase. Ünalmis et al. [14] studied the effects of different *L*/*D* ratio on cavity flow experimentally, and according to them, shock impingement effect is independent of *L*/*D* ratio and higher *L*/*D* ratios mean higher acoustic oscillations. Perng [15], Lazar et al. [16], and Stallings [17] also studied supersonic cavity flow experimentally.

Proper Orthogonal Decomposition (POD) is a method whose purpose is to obtain reduced order models of the systems by reducing the degree of the data samples collected as results of numerical or experimental studies (Cao et al. [18]). POD was introduced by Karhunen and Loéve, and this method was differently interpreted by researchers mainly with two definitions: Karhunen-Loéve decomposition *d* and *a* combination of Karhunen-Loéve decomposition, principal component analysis and singular value decomposition (Holmes et al. [19]; Chatterjee [20]; Feeny and Kappagantu [21]; Ravindra [22]; Kappagantu and Feeny [23]). Due to the compatibility of methods with each other, the second method is usually preferred by researchers (Liang et al. [24]). 

Lumley [25] and Aubry et al. [26] performed the pioneering studies using POD for fluid mechanics problems. Other studies including POD applications have been performed by researchers for cavity flows which is an important fluid flow problem. Rowley et al. [27] studied flow pass over an open rectangular cavity for different *L*/*D* ratios. They used POD to get reduced order model of the system for control applications. Nagarajan et al. [28] studied POD based modeling of an open cavity with *L*/*D* ratio of 2 and Mach number of 0.6 to perform optimal control for cavity flow. In the study of Bortz et al. [29], simulations of an open cavity with a Mach number of 0.85 and *L*/*D* ratio of 4.5 are performed for the control of cavity acoustics. Due to high storage requirements of data, also for postprocessing, Proper Orthogonal Decomposition method is used to optimize and eliminate this problem. Colonius [30], Caraballo et al. [31], and Kasnakoglu [32] are other researchers who studied POD-based models of cavity flows. Berkooz et al. [33] and Holmes et al. [19] provide a more general discussion and detailed description of POD.

The main aim of this study is to observe the effects of length to depth ratio (*L*/*D*) in an open rectangular cavity simulation for two-dimensional, supersonic turbulent cavity flow. Proper Orthogonal Decomposition (POD) method is used to further analyze the flow. The number of modes necessary to represent the cavity for flow control purposes is determined for each cavity configuration, and the results for each *L*/*D* ratio are compared to each other.

## 2. Methodology

### 2.1. CFD Methodology

The experimental cavity configuration of Kaufman et al. [34] was used as a basis for the study. Preliminary simulations are performed for *L*/*D* ratio of 5.07 to be compared with the experimental results of Kaufman et al. [34]. Parameters used are given in Table 1.

**Figure 2:**
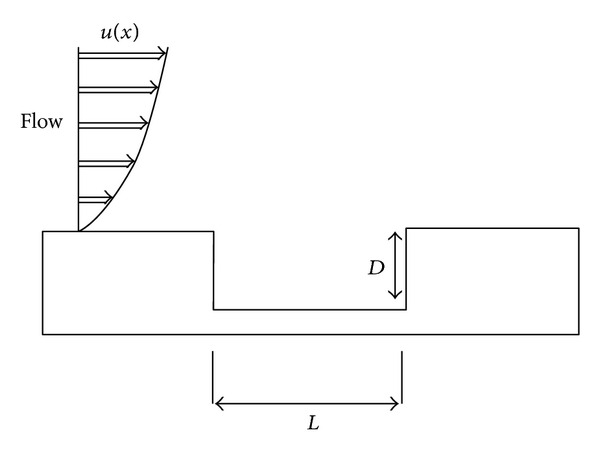
Schematic view of the cavity configuration.

In the numerical study, *k*-*ω* turbulence model is utilized. The computations are second order accurate in time and space. Two-dimensional Reynolds-averaged Navier-Stokes equations are solved. As a result of a mesh independency study, Δ*x*/*L* is 0.00062, Δ*y*/*D* is 0.00252, and average *y* ^+^ (average nondimensional cell height) is 3.4. Inflow boundary conditions are obtained from the numerical solution of two-dimensional, steady, turbulent flow over a flat plate by using the program EDDYBL (Wilcox [35]). Schematic view of the cavity geometry is given in Figure 2. The numerical boundary layer thickness for the inflow is matched with the experiment of Kaufman et al. [34]. Boundary conditions are placed far enough to avoid reflection. Pressure far field boundary condition is used for inlet, outlet, and upper wall. For other boundaries, no slip wall boundary condition is given with adiabatic wall temperature 304.8 K. Simulations are performed for 20.000 time steps. For each time step 20 inner iterations are used.

To compare the numerical pressure oscillation frequency values with Rossiter frequency values, given semiempirical equation by Rossiter [4] is used:

(1) $$\begin{array}{c} f_{m}=\frac{u_{\infty }}{L}\left[\frac{m-\alpha }{M_{\infty }+1/K_{v}}\right]. \end{array}$$

Also the modified Rossiter formulation by Heller and Bliss [5] is used to calculate frequencies:

(2) $$\begin{array}{c} \text{S}{\text{t}}_{m}=\frac{f_{m}L}{U_{\infty }}=\frac{m-\alpha }{M_{\infty }{\left(1+\left[\left(\gamma -1\right)/2\right]M_{\infty }^{2}\right)}^{-1/2}+1/K}. \end{array}$$

In (2), *K* and *α* are experimental constants. *K* is a function of Mach number and equals to 0.55 (Aradag [1]). The parameter *α* is related to cavity geometry and has a value of 0.25 (Syed [2]). *U*_*∞*_ is free-stream velocity, *M*_*∞*_ is the free-stream Mach number, St is the Strouhal number, and *m* is the mode number of the cavity.

Sound pressure levels are used for comparison of performed study with experimental results. SPL levels are obtained with the equations given in the study of Aradag [1].

Using the pressure data obtained as results of simulations, to determine dominant modes frequencies, fast Fourier transform (FFT) is used. Frequency to power spectrum graph is obtained. Additionally, *L*/*D* ratios of 1, 3, 7.6, and 10 cavity flow simulations at Mach number of 1.5 are performed. Different simulation cases based on geometrical changes are presented in Table 2.

### 2.2. POD Methodology

After a CFD simulation is performed for *M* time steps, snapshots obtained include *x*-velocity data for *M* number of time steps. *x*-velocity data in each snapshot are collected in *U*_*i*_(*x*) matrix. As a result, the following equation is obtained:

(3) $$\begin{array}{c} U_{i}\left(\vec{x}\right)=U_{1}\left(x\right),U_{2}\left(x\right),U_{3}\left(x\right),\ldots ,U_{M}\left(x\right). \end{array}$$

To eliminate requirements of scaling in further steps as defined in the studies of Newman [36] and Deane et al. [37], average of all data *n* is calculated and subtracted from each data matrix:

(4) $$\begin{array}{c} V_{i}\left(\vec{x}\right)=U_{i}\left(\vec{x}\right)-\frac{1}{M}\sum_{i=1}^{M}U_{i}\left(\vec{x}\right)\;i=1,2,\ldots ,M. \end{array}$$

To find basic functions which represent the dominant structures in the system, the function given below is used (Newman [36]):

(5) $$\begin{array}{c} \phi \left(\vec{x}\right)=\sum_{i=1}^{M}\alpha_{ik}V_{i}\left(\vec{x}\right)\;k=1,2,\ldots ,S\;\;\left(\text{mode}\;\text{number}\right). \end{array}$$

By using the method of snapshots developed by Sirovich [38], an *M* × *M* dimensional covariance matrix is obtained as (Ly and Tran [39]; Smith et al. [40])

(6a) $$\begin{array}{c} C\phi_{i}=\lambda_{i}\phi_{i}\;i=1,2,3,\ldots ,M, \end{array}$$

(6b) $$\begin{array}{c} {\left(C\right)}_{ij}=\frac{1}{M}\int_{\Omega }^{}V_{i}\left(\vec{x}\right)V_{j}\left(\vec{x}\right)dx\;i,j=1,2\ldots ,M. \end{array}$$

This covariance matrix can be solved mathematically. By using singular value decomposition, eigenvalues and eigenvectors are obtained (Volkwein [41]; Chatterjee [20]):

(7) $$\begin{array}{c} C=R\sum P^{T}. \end{array}$$

*R* contains the eigenvectors. After obtaining the eigenvalues, they are sorted starting from the largest as *λ*_1_ > *λ*_2_ > *λ*_3_ > ⋯>*λ*_*M*_. By examination of energy information, the number of modes to represent the system can be obtained (Berkooz et al. [33]).

For the reconstruction of the reduced order model, the following equation is used (Cohen et al. [42]):

(8) $$\begin{array}{c} U=\overset{-}{U}+\sum_{k=1}^{S}\alpha_{k}\phi_{k}. \end{array}$$

*U* is the original data set, $\overset{-}{U}$ is the matrix for the mean values, *α*_*k*_ are time coefficients, *ϕ*_*k*_ are basis functions, and *S* is total number of modes.

## 3. Results

### 3.1. 2D Results for Flow Structure

**Figure 3:**
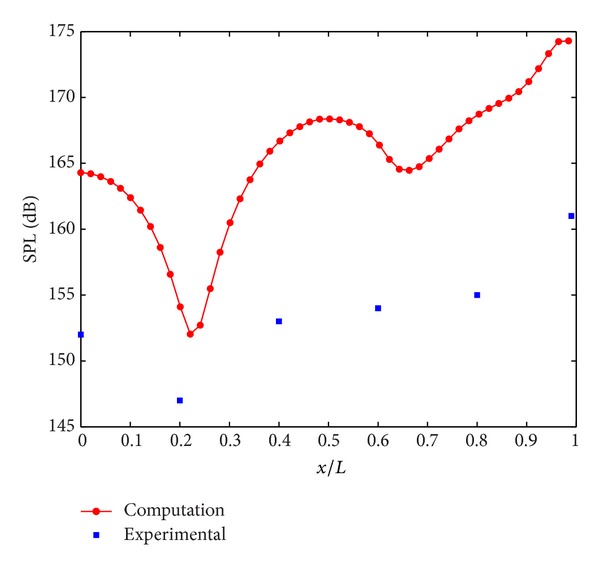
The comparison of SPL distribution at cavity floor.

As it is seen in Figure 3, the SPL values for the preliminary study which utilizes a cavity configuration with an *L*/*D* ratio of 5.07 show the same trend with the experimental results. 

**Figure 4:**
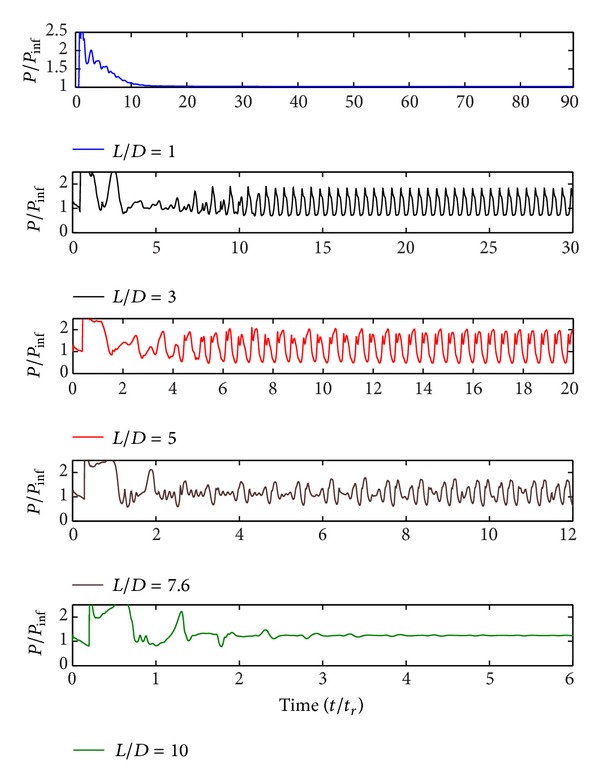
Pressure time history of the aft bulkhead at *y*/*D* = 0.6.

The flow is composed of vortex-wall, shear layer-wall, and shock waves interactions, and these interactions cause pressure oscillations. According to changes in *L*/*D* ratio, pressure oscillation mechanism also changes as it is seen in Figure 4. 

Cavity with *L*/*D* ratio of 1 is a deep cavity (Garner et al. [43]). In this configuration, due to the inactivity of the shear layer, pressure fluctuations are not observed and cavity region interactions are observed in low levels. Cavities with *L*/*D* ratios of 3, 5.07, 7.6, and 10 are all shallow cavity configurations. In cavity configurations with *L*/*D* ratios of 3, 5.07, and 7.6, shear layer separates from the leading edge of the cavity and reattaches at the back wall. Shear layer forms two flow zones as cavity flow and free-stream external flow. Since there is a pressure difference between two flow zones, mass inlet and outlet in cavity region are observed. Additionally, pressure fluctuations occur. This flow mechanism represents the open cavity flow as expected. After the flows become fully developed, periodic pressure oscillations are observed as it is seen in Figure 4. The increase in the *L*/*D* ratio triggers the pressure oscillations. The highest pressure oscillations amplitude is observed in cavity flow with *L*/*D* ratio of 5. As it is seen clearly in Figure 4, periodicity of pressure oscillations becomes irregular with the increase in *L*/*D* ratio. In cavity flow with *L*/*D* ratio of 10, the pressure fluctuations are almost damped due to being in transition flow region, between open and closed cavity flow.

**Figure 5:**
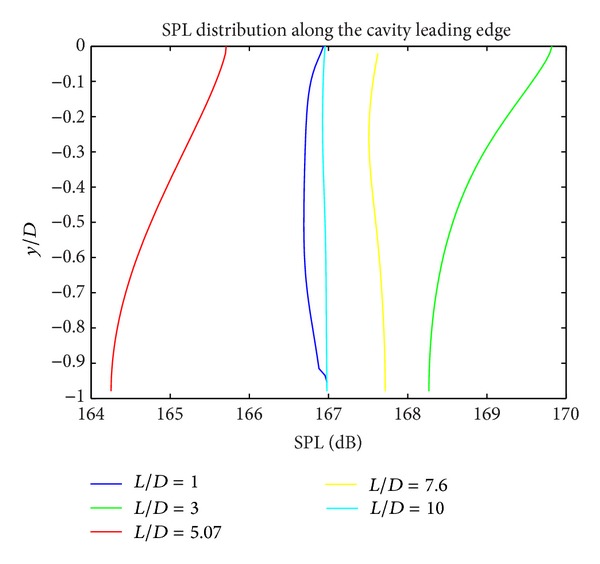
Sound pressure level distribution along the cavity leading edge.

**Figure 6:**
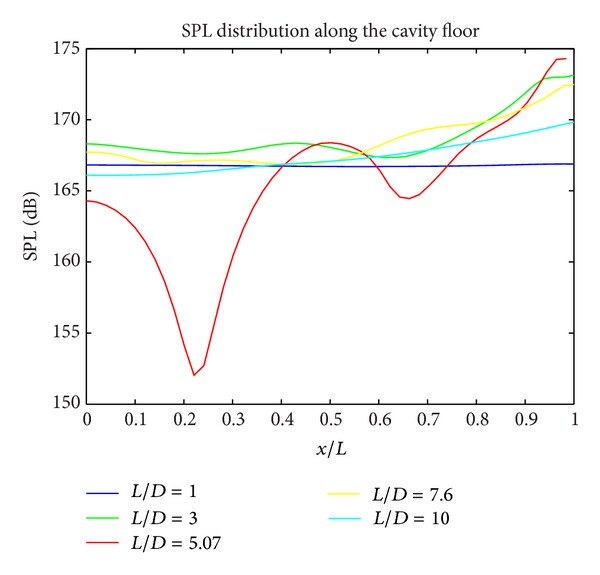
SPL distribution along the cavity floor.

**Figure 7:**
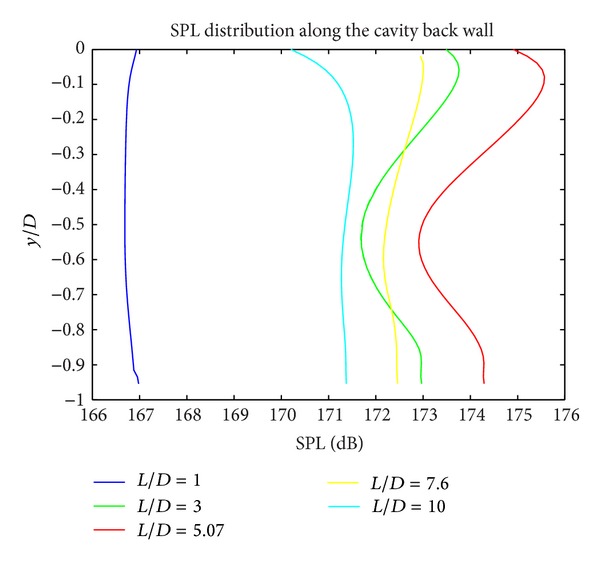
SPL distribution along the cavity back wall.

In Figures 5, 6, and 7, the acoustic pressure distributions at different regions for all cavity configurations are presented. For the cavities with *L*/*D* ratios of 1 and 10, generally lowest SPL levels are obtained. For all *L*/*D* ratios at floor edge (*x*/*L* = 1), SPL levels increase due to the interaction between shear layer and cavity wall. The highest SPL values are obtained at the cavity rear wall for all geometries. 

**Figure 8:**
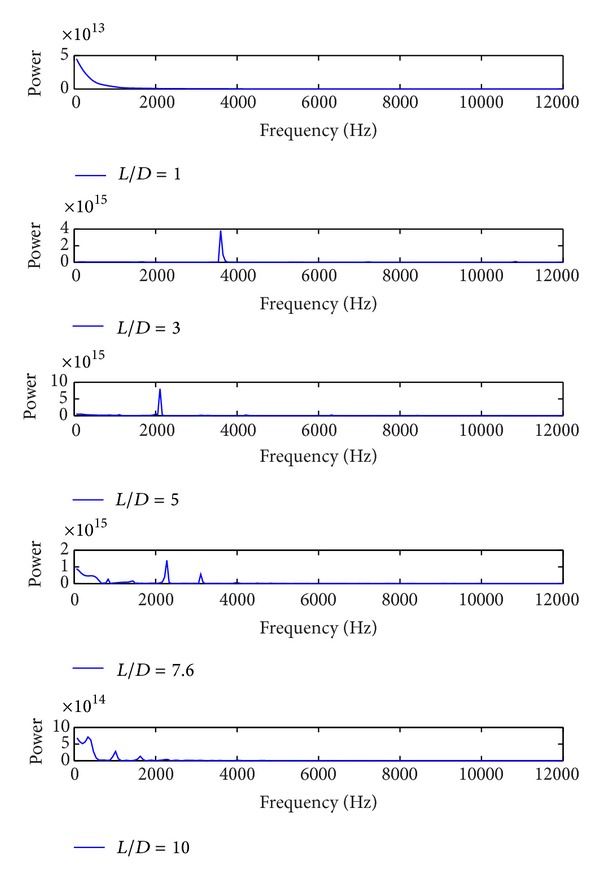
Power spectrum on the aft bulkhead *y*/*D* = 0.6.

For all cavity configurations, frequencies are calculated using the Rossiter and modified Rossiter formulations (Rossiter [4]; Heller and Bliss [5]) to be compared with the results of simulations. Fast Fourier transform is applied to the pressure results of simulations to obtain mode frequencies. As a result, frequency versus power spectrum graph is obtained from pressure history at the experimental measurement point which is located at *y*/*D* = 0.6 on the aft bulkhead. The results of FFT are given in Figure 8, and the empirical modes frequency formulations are summarized in Table 3. 

Peak powers are called dominant modes, and when first mode, is dominant, the flow has a single mode. In flows that include multiple modes, system has more than one mode and dominant frequency is seen in progressive modes. For cavities with *L*/*D* ratios of 1 and 10, pressure-time histories prove that flow is dominated by a single mode. For *L*/*D* ratio of 1, flow is influenced by a frequency of 4823 Hz, and, for the cavity flow with *L*/*D* = 10, a single mode exits equal to 388 Hz. For cavities with *L*/*D* ratios of 3, 5, and 7.6 dominant modes, which are 3880.1 Hz, 2106 Hz, and 2273 Hz, occur at second mode. When the flow has multiple modes, flow mechanism is more complicated. Results show that with the increase of the mode values, a better agreement between Rossiter formulation and computational Strouhal number values occurs. 

**Figure 9:**
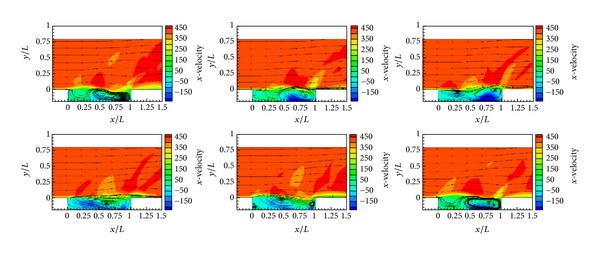
*x*-velocity contours of cavity flow with *L*/*D* ratio of 5.07.

For all cases, flow fields include a large trailing-edge vortex and two small corner vortices at rear and trailing edges of the cavity. At the cavities with *L*/*D* ratio of 1 and *L*/*D* ratio of 10, shear layer deflection is small in size, weak in strength, and no shock waves exist. In other geometries, which have multiple modes, vortex motion, shear layer deflection, and shock wave generation occur. The velocity contours of cavity flow with *L*/*D* ratio of 5 is given in Figure 9. These contours represent a clear open cavity flow mechanism using *x*-velocity data for different times along one Rossiter period.

### 3.2. POD Results for the Cavity Configuration with *L*/*D* Ratio of 5.07

**Figure 10:**
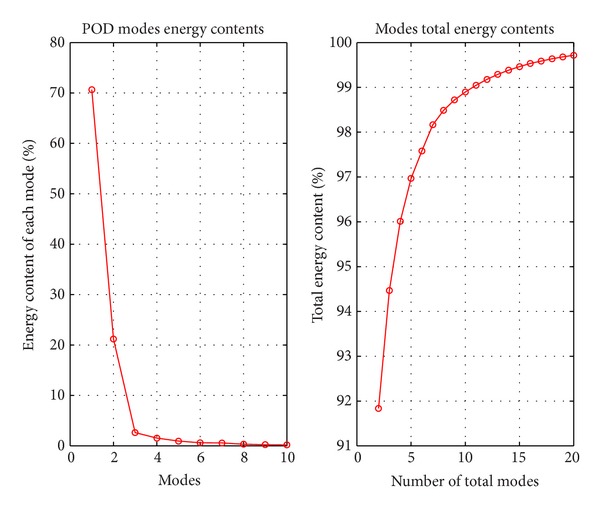
Energy distribution of modes.

As a result of POD application to the supersonic cavity flow with *L*/*D* ratio of 5.07, energy distribution is given in Figure 10 and energy contents of modes are obtained and presented in Table 4. This energy distribution is obtained by using energy contents of each eigenvalue obtained as a result of POD.

As it is seen in Table 4, the first mode includes 70.65% of total energy of the flow after which the energy content values decrease rapidly. 99.18% of the total energy of the system can be represented using 12 modes. There are many small structures which affect the main characteristics of the flow due to turbulent nature of the flow. It is clearly seen in Table 4 that there are many small turbulent structures which contain small energy values that affect the main flow. For future studies for flow control, the small structures are not important since the main idea is to control larger structures from which the smaller structures develop.

**Figure 11:**
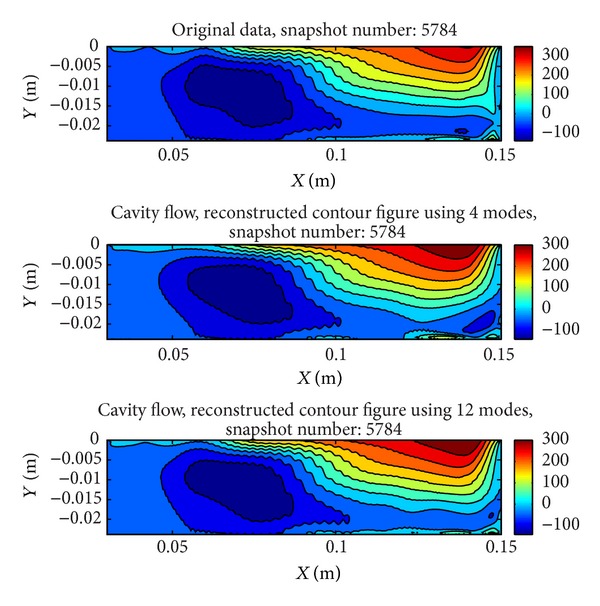
Original *x*-velocity contour and reconstructed *x*-velocity contours.

**Figure 12:**
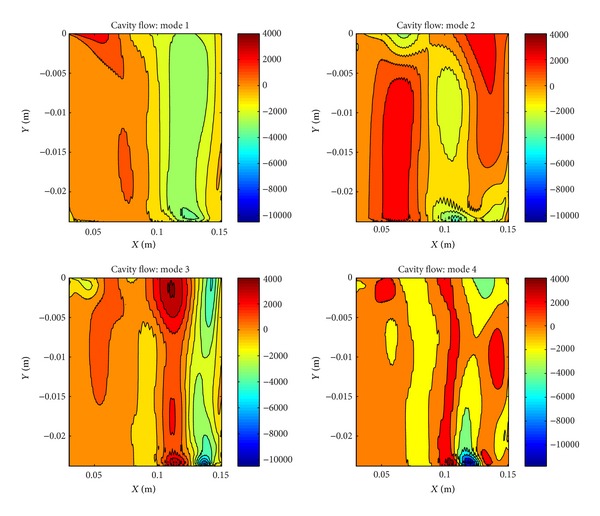
POD modes of cavity flow with *L*/*D* ratio of 5.07.

In Figure 11, comparison of original cavity contour with reconstructed cavity contours with 4 modes and 12 modes is given. There are small differences between reconstructed contours therefore the system can be represented with 4 modes which contain 96% of the total energy. In Figure 12, modes are given. As it is seen, there are some serrated structures due to the small turbulent structures.

**Figure 13:**
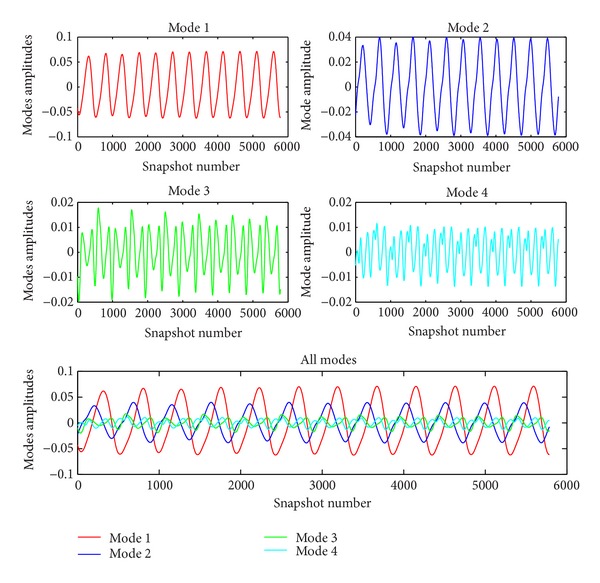
Time coefficient history of POD modes of *L*/*D* ratio of 5.07 cavity.

The dominant changes of characteristics of flow are presented with the time coefficient history of modes which is given in Figure 13. The motion of dominant structures in time can be seen clearly. The amplitudes and energy contents of modes are directly proportional to each other. As one mode has higher energy content, it also has higher mode amplitude. For Mode 1 and Mode 2, the structures show a sinusoidal periodic oscillation. Mode 3 and Mode 4 include periodical but unpredictable oscillations due to the effects of small turbulent structures.

### 3.3. The Effects of *L*/*D* Ratio on Cavity Flow Physics Based on POD Results

POD is applied to the CFD results of several cavity configurations with different *L*/*D* ratios. The necessary number of modes to represent the flow without loss of information for all cavity configurations is presented in Table 5. Systems can be represented with number of modes corresponding to 94–97% of the total system energy. 

**Figure 14:**
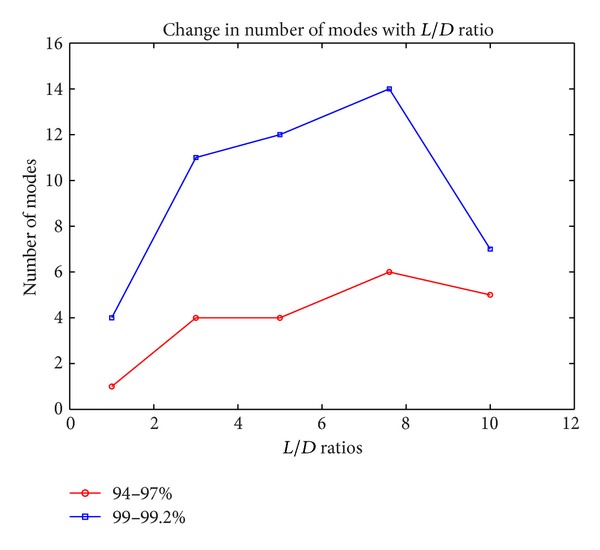
Change in the number of POD modes with *L*/*D* ratios.

As it is seen in Figure 14, with the increase in *L*/*D* ratio, the number of modes increases. Only in the cavity with *L*/*D* ratio of 10, POD results show divergence from the general trend due to being in the transition region. The cavity with *L*/*D* ratio of 1 is a deep cavity and flow interactions are low (Ayli [44]). The representation of this cavity configuration using the least number of modes shows that the flow is more uniform than others. Between the range of *L*/*D* ratios of 3 and 10, the flows become more complicated with the increase in *L*/*D* ratio, and the number of POD modes to define the systems increases with increasing *L*/*D*. 

Each mode has an energy value. The amount of these energy values is related with how the modes include characteristics of the system at hand. Energy content of each mode is given in Table 6. All cases include multiple modes except for the *L*/*D* ratio of 1. Including multiple modes means that the flow is affected by small structures. This conclusion shows good agreement with the CFD results of the same cavity configurations by Ayli [44].

## 4. Discussion and Conclusion

Supersonic cavity flow is examined to present the flow mechanism. CFD simulations are performed. Additionally, Proper Orthogonal Decomposition method is applied to the results of the CFD simulations. The effects of length to depth (*L*/*D*) ratio on the flow mechanism are investigated using CFD. Proper Orthogonal Decomposition method is applied to *x*-velocity component results of simulations, and the cavity configurations are represented with several number of modes which are available for flow control applications. With 2D cavity results, the supersonic cavity flow mechanism is represented for different cavity configurations. POD results and CFD results show good agreement with each other. As a result of Computational Fluid Dynamics simulations and Proper Orthogonal Decomposition,

- in the range of *L*/*D* ratios of 3 and 10, the exact open cavity phenomenon is observed. As the *L*/*D* ratio increases, regularity of periodic structures are decreased, and flow becomes more complicated. As a result of fast Fourier transform, it is shown that these flows include multiple modes. The number of POD modes to represent these systems also increases as *L*/*D* ratio increases.
- In the cavity with *L*/*D* ratio of 1, a low level of flow structure interactions is observed and almost no pressure oscillations occur. The flow is not as complex as the others and this is supported by POD results. The least number of POD modes necessary to represent the system is observed in this case. In the cavity with *L*/*D* ratio of 10, nearly no pressure oscillations are observed as in the *L*/*D* = 1 cavity. However, POD results show that this flow is not as simple as in *L*/*D* = 1 cavity flow This *L*/*D* ratio is in the range of transition region between open cavity and closed cavity; therefore the behavior of the flow is not as clear as others.
- In all cases, POD results show that the modes including small energy values affect the main flow. This can be expressed with the effects of small turbulent structures on the main flow.

For flow control phenomena, the control of the larger structures is the main objective. Therefore, in this study, it is shown that supersonic cavity configurations can be represented with several POD modes that include 95–97% of the total energy of the flow. For flow control purposes, these large scale structures represented by first several modes can be targeted, and small scale fluctuations may not exist anymore since they develop from the larger ones.

## Figures and Tables

**Table 1:** Numerical and experimental parameters for the preliminary CFD study.

Total pressure	66.4 kPa
Total temperature	218 K
Mach number	1.5
Reynolds number	1.09 × 10^6^
Cavity length	0.12065 m
Cavity depth	0.0238 m
Boundary layer thickness	0.0051 m

**Table 2:** *L*/*D* ratios of cavities.

	*L* (m)	*D* (m)
*L*/*D* = 1	0.0238	0.0238
*L*/*D* = 3	0.0714	0.0238
*L*/*D* = 5.07	0.12065	0.0238
*L*/*D* = 7.6	0.180975	0.0238
*L*/*D* = 10	0.2413	0.0238

**Table 3:** Experimental and calculated mode frequencies.

*L*/*D*	Frequency modes	Computational values	Modified Rossiter form.	Rossiter form.	Discrepancy (%)	Discrepancy Rossiter form. (%)
1	*f*1	4823.0	4566.7	5622.2	5.6	14.2
1	*f*2	10034.0	10656.0	11244.4	5.8	10.7
1	*f*3	—	16744.0	16866.6	—	—
3	*f*1	1940.0	1522.2	1663	27.0	16.7
3	*f*2	3880.1	3551.8	3659	9.2	6.0
3	*f*3	5559.2	5581.5	5488	0.4	1.3
5	*f*1	1037.0	900.8	1109.1	15.0	6.5
5	*f*2	2106.0	2102.0	2218.0	0.19	5.0
5	*f*3	3160.0	3303.1	3327.0	4.5	5.0
7.6	*f*1	609.8	600.55	739.4	1.5	17.5
7.6	*f*2	1441.0	1401.3	1478.7	2.8	2.5
7.6	*f*3	2273.0	2202.1	2218.1	3.2	2.5
10	*f*1	388.0	450.41	554.5	13.7	30.0
10	*f*2	1053.0	1051.0	1109.1	0.19	5.1
10	*f*3	1663.0	1651.5	1663.5	0.71	0.1

**Table 4:** *L*/*D* = 5 cavity, POD modes energy contents.

*L*/*D* = 5, cavity configuration												
Mode number	1	2	3	4	5	6	7	8	9	10	11	12
Energy content % (/96 )	70.65	21.29	2.63	1.54	—	—	—	—	—	—	—	—
Energy content % (/99)	70.65	21.29	2.63	1.54	0.96	0.61	0.58	0.32	0.23	0.18	0.15	0.13

**Table 5:** The necessary number of POD modes for all cases.

Cavity configurations	*L*/*D* = 1		*L*/*D* = 3		*L*/*D* = 5.07		*L*/*D* = 7.6		*L*/*D* = 10	
Total Energy content %	94.33	99.41	95.58	99.17	96	99.18	95.33	99.19	96.82	99.18
Number of modes	1	4	4	11	4	12	6	14	5	7

**Table 6:** Energy content of each mode for all cavity configurations.

Energy content of each mode for all cavity configurations %										
Mode number	*L*/*D* = 1		*L*/*D* = 3		*L*/*D* = 5.07		*L*/*D* = 7.6		*L*/*D* = 10	
1	94.33	94.33	67.02	67.02	70.65	70.65	60.87	60.87	46.38	46.38
2	—	2.88	20.60	20.60	21.19	21.19	18.15	18.15	31.06	31.06
3	—	1.64	4.86	4.86	2.63	2.63	7.12	7.12	12.15	12.15
4	—	0.56	3.10	3.10	1.54	1.54	5.17	5.17	4.2	4.2
5	—	—	—	0.090	—	0.96	2.16	2.16	3.02	3.02
6	—	—	—	0.078	—	0.61	1.86	1.86	—	1.67
7	—	—	—	0.067	—	0.58	—	1.14	—	0.69
8	—	—	—	0.048	—	0.32	—	0.71	—	—
9	—	—	—	0.030	—	0.23	—	0.55	—	—
10	—	—	—	0.0026	—	0.18	—	0.40	—	—
11	—	—	—	0.0020	—	0.15	—	0.36	—	—
12	—	—	—	—	—	0.13	—	0.30	—	—
13	—	—	—	—	—	—	—	0.23	—	—
14	—	—	—	—	—	—	—	0.17	—	—

Total energy content %	94.33	99.41	95.58	99.17	96.01	99.18	95.33	99.19	96.82	99.18
